# Multimodal X-ray imaging of nanocontainer-treated macrophages and calcium distribution in the perilacunar bone matrix

**DOI:** 10.1038/s41598-020-58318-7

**Published:** 2020-02-04

**Authors:** Karolina Stachnik, Martin Warmer, Istvan Mohacsi, Vincent Hennicke, Pontus Fischer, Jan Meyer, Tobias Spitzbart, Miriam Barthelmess, Jacqueline Eich, Christian David, Claus Feldmann, Björn Busse, Katharina Jähn, Ulrich E. Schaible, Alke Meents

**Affiliations:** 1 DESY Photon Science, Deutsches Elektronen-Synchrotron DESY, Hamburg, 22607 Germany; 2 Center for Free-Electron Laser Science, Hamburg, 22607 Germany; 3 Research Center Borstel – Leibniz Lung Center, Borstel, 23845 Germany; 4 Paul Scherrer Institute, Villigen, PSI 5232 Switzerland; 5 Institute of Inorganic Chemistry, Karlsruhe Institute of Technology (KIT), Karlsruhe, 76131 Germany; 6 Department of Osteology and Biomechanics, University Medical Center Hamburg-Eppendorf, Hamburg, 22529 Germany

**Keywords:** Nanoscience and technology, Optics and photonics, Biophysics

## Abstract

Studies of biological systems typically require the application of several complementary methods able to yield statistically-relevant results at a unique level of sensitivity. Combined X-ray fluorescence and ptychography offer excellent elemental and structural imaging contrasts at the nanoscale. They enable a robust correlation of elemental distributions with respect to the cellular morphology. Here we extend the applicability of the two modalities to higher X-ray excitation energies, permitting iron mapping. Using a long-range scanning setup, we applied the method to two vital biomedical cases. We quantified the iron distributions in a population of macrophages treated with *Mycobacterium-tuberculosis*-targeting iron-oxide nanocontainers. Our work allowed to visualize the internalization of the nanocontainer agglomerates in the cytosol. From the iron areal mass maps, we obtained a distribution of antibiotic load per agglomerate and an average areal concentration of nanocontainers in the agglomerates. In the second application we mapped the calcium content in a human bone matrix in close proximity to osteocyte lacunae (perilacunar matrix). A concurrently acquired ptychographic image was used to remove the mass-thickness effect from the raw calcium map. The resulting ptychography-enhanced calcium distribution allowed then to observe a locally lower degree of mineralization of the perilacunar matrix.

## Introduction

Metal ions play an important role in the vital functions of living organisms. They are present in various biological systems in a vast range of concentrations as structural, electrolyte (minor), and trace elements. From being major tissue components (e.g. Ca in bones) to constituents of essential biological molecules (e.g. Fe in hemoglobin), metals take part in the majority of extra- and intracellular processes. In particular, first-row transition metals (Mn, Fe, Cu, Ni, Zn) – despite their minute concentrations – are involved in sub-cellular processes. Moreover, their abnormal accumulation in human brain was correlated with mechanisms leading to neurodegeneration diseases such as Parkinson’s and Alzheimer’s^1–3^. Metal compounds have also been utilized as novel drug delivery systems by addressing metabolic properties of bacterial agents^4,5^ or as more efficient medical imaging markers tracking tissue of interest^6–8^.

In all aforementioned cases, studies of metal contributions require knowledge of their quantitative spatial distributions with respect to the sub-cellular structure. Electron-probe Energy-Dispersive Spectroscopy offers elemental mapping at nanometer-range spatial resolutions and high excitation efficiency in the low-Z-element range. Yet, it is vastly limited by short penetration depth, Bremsstrahlung background from the electron source, reducing peak-to-background ratio, and invasive sample preparation requirements^9^. On the contrary, recent developments in hard X-ray microscopy at modern synchrotron radiation sources have opened new paths of non-destructive and diverse probing of biological specimens. With advances in hard X-ray nanofocussing, nanoscale X-ray fluorescence (XRF) can provide a unique elemental contrast at below 100-nm spatial resolutions and remarkable excitation efficiency of elements in trace concentrations. Yet, interpretation of XRF data may become challenging due to missing information on morphology of a measured biological specimen. Enhanced coherence properties and higher photon fluxes of third-generation synchrotrons have promoted phase contrast hard X-ray imaging techniques for the investigation of weakly absorbing biological structures. Combination of propagation-based full-field phase contrast imaging and X-ray fluorescence mapping was used in quantitative elemental analysis of single cells^10^ and extended tissue sections^11^. The method of scanning Zernike phase contrast was implemented along with XRF to qualitatively correlate the occurrence of low-Z elements with sub-cellular organelles^12^. Over the last decade, ptychographic coherent diffractive imaging^13,14^ has been established as a robust scanning X-ray microscopy method. It provides quantitative optical density contrast at dose-limited spatial resolutions beyond the fabrication limits of X-ray optics. It uses an iterative phase retrieval algorithm^15–17^ to reconstruct complex object and probe functions from a redundant set of far-field diffraction patterns. The redundancy is achieved by keeping a known relative spatial overlap^18^ between illuminated areas. Thanks to its sensitivity, the technique is suitable for imaging of both radiation-resistant high-Z-element-rich specimens and soft biological tissue allowing for a vast range of applications. It can exploit both chemical^19–22^ and magnetic^23,24^ contrasts.

In the advent of diffraction-limited synchrotron light sources^25^, ptychography was consolidated with nanoscale XRF in concurrent imaging of a freshwater diatom^26^. Both techniques were further extended by in-vacuum cryogenic sample cooling, providing simultaneously qualitative distributions of light elements within a cell and its morphology at remarkable sensitivity and sub-50-nm spatial resolution^27^. Further developments involved the implementation of a continuous-motion scanning scheme^28–31^ and a sample rotation which enabled simultaneous acquisition of a tomographic dataset in both modalities with reduced time overhead^32^. Yet, these demonstrated solutions have until now featured an accessible field of view limited to the imaging of single cells. Recently, quantitative correlative 2D imaging of five entire nematodes has been demonstrated using a 2.5-μm beam (FWHM) and typical X-ray fluorescence microscopy scan parameters for scanning areas of up to 0.1 mm^2^ at an energy of 10 keV, though with a resolution of 280 nm in ptychography^33^.

However, reaching higher spatial resolutions in both modalities has so far been restrained by insufficiently coherent flux at higher photon energies. As a result, the concurrent ptychographic and nanoscale X-ray fluorescence imaging has practically allowed only for low-Z-element mapping in single specimens with X-ray excitation energies limited to 5.2 keV. This limitation has until now stemmed from relatively high emittance values of synchrotron sources that kept the spatially coherent fraction of X-ray beam below 0.1% at high photon energies (>10 keV). New low-emittance storage rings, such as PETRA III or NSLS-II, and recently emerging diffraction-limited synchrotron sources (MAX IV) allow to overcome this limitation, providing up to 2 orders of magnitude improvement in the degree of spatial coherence^25^. Hence, this latest generation of synchrotron sources brings advances in quantity, enabling the increase of experimental throughput and statistical relevance, as well as in quality, such as enhancement in signal-to-noise ratio and spatial resolution^34^.

Characteristics of ultralow-emittance synchrotron storage rings have triggered substantial development in fast scanning approaches, permitting an efficient use of the augmented X-ray flux. Adaptation of the continuous-motion scanning in ptychography and the mixed-state reconstruction algorithm^35^ have certainly rendered a prospect of the considerable decrease of measurement time overhead. Yet, the so-far proposed piezo scanning units have offered an effective scan area of around 100 × 100 μm^2^ with an interferometric control of motor positions^36–40^. While it is sufficient for imaging of single cells and confined tissue volumes, scanning larger areas is still prone to artefacts induced by a coarse positioning system^41^. In order to overcome these limitations we have developed a flexure-based piezomotorized stage, allowing for fast scanning of an area of 4 × 4 mm^2^.

We have further extended the multimodal concept of probing biological specimens with simultaneous ptychography and nanoscale X-ray fluorescence to higher X-ray energies. Here we demonstrate the application of this method in correlative imaging of macrophages treated with Fe_2_O_3_ nanocontainers, that target antibiotic drugs to intracellular *Mycobacterium tuberculosis*, and in a study of bone matrix mineralization proximal to osteocyte lacunae.

## Iron-Oxide Nanocontainers in Macrophages

Innovative nano-medical approaches for drug delivery aim at enhancing local and reducing peripheral drug concentrations. Iron-oxide nanocarriers^4^ represent a novel tool for a targeted delivery of the anti-tuberculosis antibiotics to infected macrophages. Tuberculosis (TB) is one of the most important bacterial infections worldwide causing high mortality and morbidity. The causative agent, *Mycobacterium tuberculosis*, is a facultative intracellular pathogen which can survive and grow in phagosomes upon phagocytosis by macrophages^42,43^. These host cells, otherwise well-equipped to kill bacterial invaders, serve as niches for mycobacteria. More importantly, the membrane-enclosed phagosome and the lipid-rich cell wall of the mycobacteria pose a significant challenge for an efficient drug delivery as they hinder antibiotics entering the bacteria. A potential solution to this inherent problem of the TB treatment are 18-nm-diameter hollow Fe_2_O_3_ nano-spheres loaded with antibiotics, which can target anti-TB drugs to intracellular mycobacteria. The nanocontainers are actively internalized into macrophages and release antibiotics in close proximity to the mycobacteria. This happens upon slow metabolic dissolution of the Fe_2_O_3_ wall, exploiting the mycobacteria’s need for iron. We used simultaneous X-ray fluorescence and ptychography to obtain iron distribution maps and structural images of nanocontainer-treated macrophages, which allowed us to quantify the amount of antibiotic delivered to the cells.

### Nanocontainer uptake

**Figure 1:**
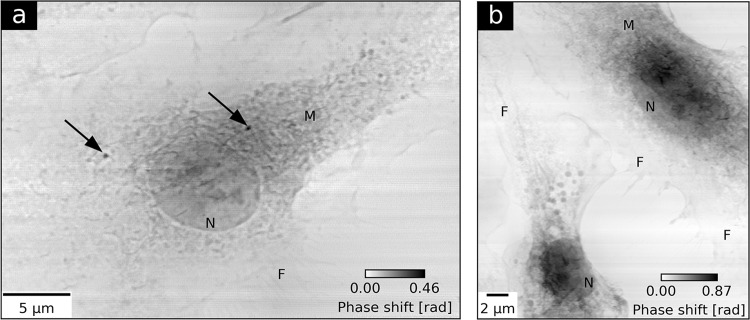
Ptychographic imaging of two groups of macrophage cells. (**a**) presents the reconstructed phase of a macrophage treated with Fe_2_O_3_ nanocontainers targeting *Mycobacterium tuberculosis*. Black arrows indicate the internalization of two agglomerates of nanocontainers in the cell. (**b**) shows the ptychographic phase of two untreated control cells. Both images allow for identification of cellular nuclei (N), membrane ruffles (M) and filopodia (F).

**Figure 2:**
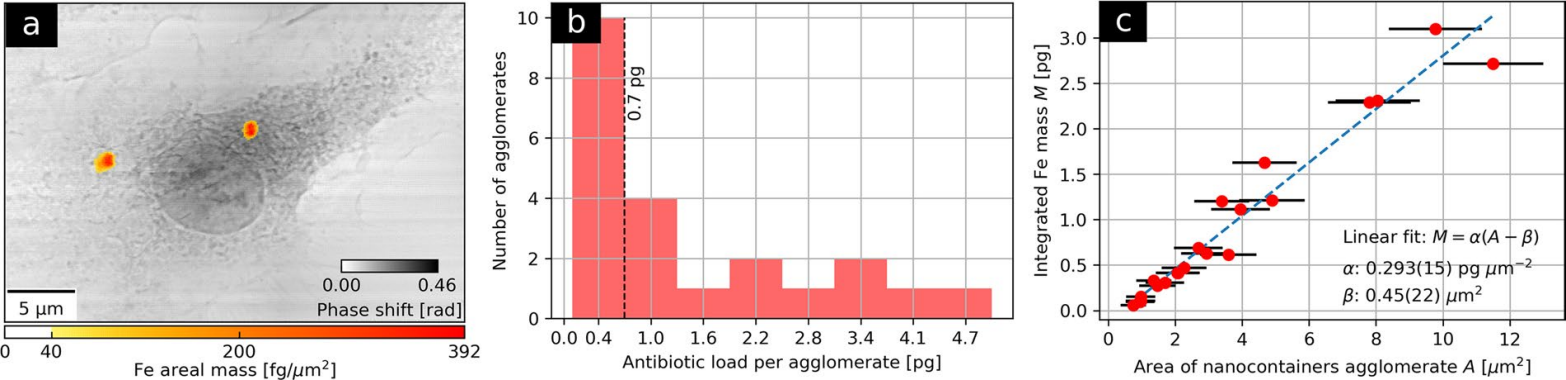
Simultaneous ptychography and X-ray fluorescence (XRF) of macrophages treated with Fe_2_O_3_ nanocontainers targeting *Mycobacterium tuberculosis*. (**a**) presents Fe areal mass map obtained by means of XRF superimposed on the ptychographic phase of a representative nanocontainer-treated macrophage. In 14 cells measured under the same conditions, 22 agglomerates of nanocontainers were found. For each of them, its integrated Fe mass and its area were calculated. The integrated Fe masses were further recalculated into the corresponding antibiotic contents in the agglomerate, using weight proportions known from the previous study^4^. (**b**) shows histogram of the estimated antibiotic load per agglomerate. Antibiotic contents below 0.7 pg tend to dominate. (**c**) presents a linear relation between the integrated Fe masses of nanocontainer agglomerates and their areas.

 Figure 1 shows ptychographic phase images of OsO_4_-stained macrophage cells. The quantitative grayscale contrast denotes a relative phase shift which is proportional to the projected electron density of a cell. In Fig. 1a, a representative macrophage treated with Fe_2_O_3_ nanocontainers is shown. The image allows to identify basic cellular structures like the nucleus (N), cell membrane ruffles (M), and macrophage-specific filopodia (F). The two high density spots, indicated with black arrows, are two nanocontainer agglomerates internalized within the cytosol. For comparison Fig. 1b shows the cytoplasm of two untreated control cells. Simultaneously to ptychographic imaging, Fe spatial distribution maps were obtained by means of X-ray fluorescence measurements. Detailed experimental parameters are provided in Table 1 (Macrophages). A 470-nm-thick iron film deposited with electron-beam evaporation on a Si_3_N_4_ membrane was used for calibrating the Fe K-line XRF yield to Fe areal mass (Supplementary Method 1). Figure 2a shows the Fe areal mass distribution map superimposed on the ptychographic phase of the nanocontainer-treated macrophage. Two distinct iron spots coincide well with the positions of the nanocontainer agglomerates obtained by ptychography. The difference in the size of the agglomerates obtained by both imaging techniques stems from the inherently superior spatial resolution offered by ptychography. The calibrated Fe map allowed to specify the maximum Fe areal masses of the agglomerates and quantify their integrated Fe masses and their areas (Table 2). The detailed calculation steps are described in Supplementary Method 1.

**Table 1:** Experimental parameters of the simultaneous ptychography and X-ray fluorescence at beamline P11.

Parameter	Macrophages	Bones
Energy [keV]	7.35	7.15
Exposure time [ms]	150	100
Detector	Pilatus 1M	Pilatus 300k
Reconstructed probe size (h × v) [nm^2^]	400 × 600	200 × 400
XRF pixel size [nm]	125	200
Ptychographic pixel size [nm]	21	33
Incident photon flux [photon s^−1^]	5.4 × 10^8^	3.7 × 10^8^
Total dose [MGy]	6.84	8.65

**Table 2:** Quantitative analysis of two nanocontainer agglomerates internalized in the macrophage in Fig. 2a.

Parameter	Left agglomerate	Right agglomerate
Maximum Fe areal mass [fg μm^−2^]	392 (18)	351 (17)
Integrated Fe mass [fg]	327 (4)	411 (5)
Area [μm^2^]	1.4 (5)	2.1 (6)
Antibiotic load per agglomerate [pg]	0.52 (5)	0.66 (6)

### Antibiotic load and mean nanocontainer concentration

For more statistically-relevant results, we measured under the same conditions a population of 14 cells, in which 22 agglomerates of nanocontainers were identified. For each agglomerate its integrated Fe mass was calculated.

Every nanocontainer comprises 43 wt% of Fe_2_O_3_ (sphere wall), 48 wt% of antibiotics, and 9 wt% of water^4^. By expressing the integrated Fe masses in terms of total Fe_2_O_3_ mass, we used the weight proportions to estimate the distribution of antibiotic load per agglomerate as shown in Fig. 2b. For almost half of the investigated agglomerates this number does not exceed 0.7 pg antibiotic per agglomerate. It is also the case for the two agglomerates shown in Fig. 2a, whose estimated antibiotic loads are provided in Table 2.

Subsequently, for each agglomerate the corresponding area was calculated. Figure 2c shows a correlation between the integrated Fe mass *M* and the area *A* of all nanocontainer agglomerates as derived from X-ray fluorescence maps. We observed a monotonic growth of the agglomerate integrated Fe mass with its area. We chose a linear test function to approximately model the observed relation. The implied average planar spread of agglomerates could potentially be related to the preparation method of the nanocontainer-treated macrophages on a flat substrate. A linear trend line was fitted according to the equation *M* = *α*(*A* − *β*) to account for the observed non-zero *A*-intercept. The linear parameter *α* of 0.293(15) pg μm^−2^ can be interpreted as a mean Fe areal mass of the entire set of agglomerates. It can also be converted into a mean number of nanocontainers (NC) per unit area of 31700(6800) NC per μm^2^. The calculation steps for expressing Fe areal masses as nanocontainer numbers are based on the known nanocontainer dimensions^4^ and are provided in Supplementary Method 1. The additive parameter *β* equals to 0.45(22) μm^2^ and can in turn be attributed to a systematic overestimate of the agglomerate areas due to the lower resolving power of XRF. This argument is also supported by visibly smaller agglomerate sizes in the ptychographic image.

### Discussion

In this application, the method of concurrent ptychography and XRF allowed to visualize and quantify the uptake of Fe_2_O_3_ nanocontainers in a population of macrophages in the context of their sub-cellular organelles. Fe maps revealed the presence of nanocontainer agglomerates while the simultaneously acquired ptychographic images complemented these with detailed cellular morphology views at sensitivity and spatial resolution unmatched by any other scanning X-ray microscopy technique. Further, the quantitative Fe areal mass maps of the nanocontainer-treated macrophages allowed to compute the distribution of antibiotic load per agglomerate. It can serve as a complementary assessment of the nanocontainer efficacy in tuberculosis infection treatment. The long penetration depth of hard X-rays allows to look beyond the sample surface and investigate the whole volume of the cell without a need of sectioning. Despite still missing depth information, the linear model of the relation between the integrated Fe mass and the area of the nanocontainer agglomerates yields an averaged number of nanocontainers per agglomerate unit area. The obtained value of 31700(6800) NC per μm^2^ remains in good agreement with the previous study based on the statistical evaluation of transmission electron microscopy images^4^.

## Bone Matrix Mineralization

Pathologies of the skeleton are among the most common morbidities worldwide. Age-related bone loss (osteoporosis) is supposed to increase in its clinical significance with the current demographic development. Its detrimental result are fragility fractures that remain difficult to treat. Bone quality is a result of the complexity of the bone micro- and nano-structure and the tissue remodelling process. The cellular network of the osteocytes in bone has emerged as key factor of bone remodelling executed by bone-resorbing osteoclasts and bone-forming osteoblasts. It has been demonstrated that the osteocytes govern a local volume of matrix surrounding the osteocyte lacunae (perilacunar matrix), which is actively turned over by the osteocytic activity^44–46^. Osteocytes acidify their lacuno-canalicular volume to demineralize the local bone matrix^44^. This mechanism appears to serve as a response to an increased calcium demand, e.g. during lactation. Yet, mechanistic studies show that osteocytes utilize a far less acidic pH than osteoclasts to dissolve the bone mineral^47^. Therefore, the perilacunar matrix would need to be more susceptible to the demineralization by means of a differential elemental or structural composition. Perilacunar matrix governed by osteocytes is hypothesized to be distinctively different from the remaining bone matrix. It is supposed to allow demineralization by the osteocytic bone resorption and easier lacuna-shape adaptation in differential loading scenarios. We have studied the spatial distribution of Ca concentration in an unstained and resin-embedded thin human bone section using simultaneous ptychographic and X-ray fluorescence measurements.

**Figure 3:**
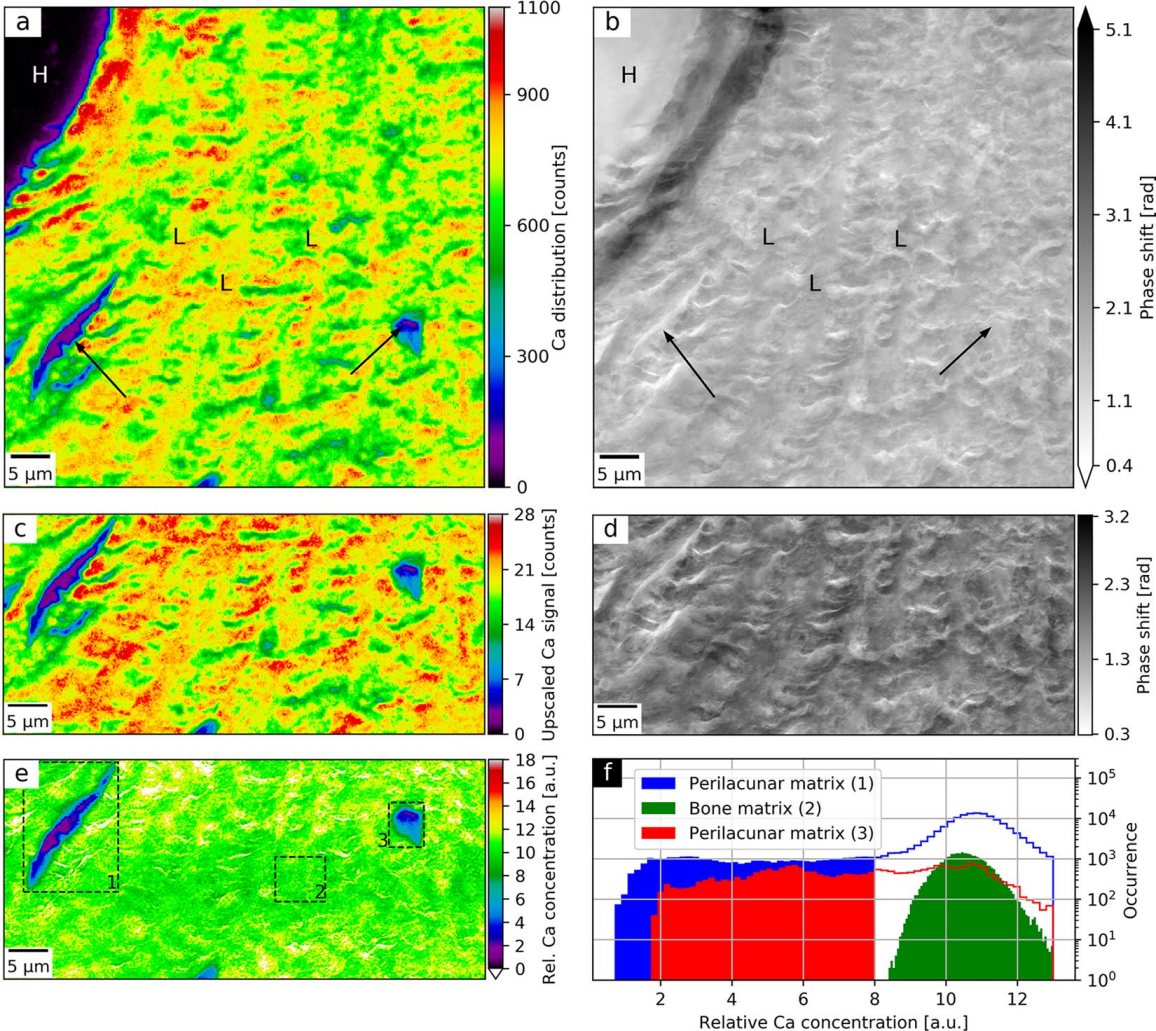
Spatial distribution of relative Ca concentration of a human bone section using simultaneous ptychography and X-ray fluorescence (XRF) at 7.15 keV. (**a**) shows the Ca distribution as obtained by XRF mapping and (b) the ptychographic phase shift. Both (**a**,**b**) allow to identify a Haversian canal (H) and concentric lamellae (L). In (**a**), the Ca depletion areas (black arrows) indicate perilacunar matrices of two osteocyte lacunae, while the corresponding areas in (**b**) exhibit no change in phase shift, which is proportional to the projected density. The lower half of the Ca map was bilinearly upscaled (**c**) and divided by the regularized ptychographic phase (**d**). (**e**) presents the mass-thickness-corrected Ca map corresponding to the relative Ca concentration. Sample preparation artefacts were masked in white. (**f**) shows histograms of relative Ca concentrations values of three bone matrix areas as marked with dashed rectangles in (**e**). It compares relative Ca concentrations between two regions enclosing the identified perilacunar matrices (1, 3) and a representative region of the bone matrix (2). Face-filled in blue and red histogram values correspond to actual areas of the perilacunar matrices and span over visibly lower range of relative Ca concentrations than the values from the bone matrix (in green).

We measured a 70 × 70 μm^2^-area of the cortical bone matrix in the proximity of a Haversian canal. Detailed experimental parameters are provided in Table 1 (Bones). Figure 3 shows the Ca map (a) and the ptychographic phase (b) of the selected bone region at spatial resolutions of 400 nm and 65 nm (Supplementary Method 2), respectively. It is possible to identify a fragment of the resin-filled Haversian canal (H), concentric lamellae rings (L), and two osteocyte lacunae (black arrows). The surrounding bone matrix was partially affected by typical cutting artefacts during sample preparation causing ruptures without any embedding medium.

### Ptychography-enhanced Ca distribution

The raw Ca map is intrinsically affected by the projected density and thickness of the specimen called the mass-thickness effect. In the absence of calibration standards, the quantitative phase contrast image acquired simultaneously with the elemental map allows for a robust correction of this distortion, free of any scanning artefacts. Therefore, the lower halves of Fig. 3a,b were selected for further correction of the mass-thickness effect as described in^11^. Since a background-corrected area under the Ca K-line peak is proportional to Ca areal mass and a ptychographic phase shift is proportional to the projected mass per unit area of the sample, the ratio of the two represents a relative Ca concentration map. The bilinearly upscaled Ca map (Fig. 3c) and the ptychographic phase (Fig. 3d) were aligned using subpixel image registration^48^. The upscaled Ca map was then divided by the ptychographic phase shift augmented by a regularization phase offset of 0.1 rad to avoid division by zero. Figure 3e shows the mass-thickness-corrected Ca map which corresponds to the relative Ca concentration. In the rupture areas stemming from cutting artefacts, the operation resulted in artificially elevated relative Ca concentrations. They originate from the difference in spatial resolution and sensitivity between XRF and ptychographic measurement. These areas were masked in white and excluded from the analysis.

### Ca depletion in the perilacunar matrix

The obtained relative Ca concentration map (Fig. 3e) was used to compare the Ca content around two osteocyte lacunae with respect to the surrounding bone matrix. For this purpose, the relative Ca concentration values of the two perilacunar matrix regions (denoted as 1 and 3 in Fig. 3e) and a representative bone matrix area (2) were compared in the histogram in Fig. 3f. In the case of regions (1) and (3), the face-filled histogram regions represent the actual areas of the perilacunar matrices. The histogram shows depletion of Ca in the two regions around the osteocyte lacunae with respect to the remaining bone matrix area.

### Discussion

In this application, simultaneous ptychography and XRF were utilized to map the Ca concentration in human cortical bone tissue. The human bone consists to a large extent of a form of hydroxyapatite making Ca the major structural element of the bone. Although both imaging modalities allow for a general structure identification, XRF analysis benefits here from the simultaneously obtained ptychographic phase which removes the mass-thickness effect. The concurrent acquisition frees both measurements from any relative image distortions. The resulting relative Ca concentration map presents a much more uniform Ca distribution than the raw Ca map. The same effect had also been observed in similar previous studies of single cells^10^ and neuronal tissue^11^. Within single micrometers around the osteocyte lacunae the Ca content visibly decreases. The comparison with concentrations in the remaining bone matrix further indicates a locally lower degree of mineralization in the perilacunar matrix^49^. Yet, a statistically-relevant population of lacunae must be investigated to address the previous studies reporting a systematically higher mass densities of the perilacunar matrix^50,51^. As an additional improvement, a local increase of the spatial resolution of Ca maps could diminish the artefacts from division by the ptychographic phase. Such higher resolutions could, for example, be realized by utilizing multilayer Laue lenses as a probe-forming focussing optic, able to focus efficiently X-ray beam into a sub-10-nm focus^52^.

## Conclusions

The work presented here demonstrates the semiquantitative, structural investigation of elemental distributions in two vital biological systems at the nanoscale. It enables high-throughput and seamless measurements of many targets with a long-range scanning unit and a highly coherent and intense X-ray beam. In this way, it extends simultaneous ptychography and X-ray fluorescence to the multimodal imaging of a statistically-relevant number of samples at higher photon energies. The method provides two complementary and quantitative contrast mechanisms. It demonstrates its potential in studying anti-tuberculosis drug delivery to macrophages, allowing for correlative measurements of the largest population of specimens to date. We further show that the method is very well suited to investigate the mineralization of human bone matrix. This work is therefore an important step towards concurrent studies of first-row transition metals distributions and the morphology of biological tissues by means of scanning X-ray microscopy.

An inherent limitation of the proposed imaging method is the lack of depth information which hinders unambiguous interpretation of superimposed features in 2D projections, especially in the case of thicker cells. Moreover, in the case of nanocontainer-treated macrophages only a complementary imaging method currently allows to distinguish whether the nanocontainer agglomerates are certainly internalized inside the cells (Supplementary Method 3). The solution is to combine both ptychography and X-ray fluorescence with computed tomography which would provide quantitative elemental and electron density contrasts of the reconstructed volume. Such an upgrade must also involve more efficient XRF detection, that will enable shorter exposure times at no loss in signal-to-noise ratio. It would also lower the dose imparted on the specimen per 2D projection, which in both reported experiments ranged from 6.85 to 8.65 MGy (Table 1). Despite no observed radiation damage, these values approach the maximum tolerable doses for room-temperature imaging of chemically fixed samples at sub-100-nm spatial resolutions^53,54^. Therefore, extending this imaging method to tomography requires implementation of a cryogenic sample cooling to reduce the radiation damage at the nanoscale level. Despite the first proof-of-principle demonstrations^32,55^, new concepts are sought to improve the robustness of such a 3D elemental and structural mapping and make it reach the limit imposed by the available coherent flux.

Simultaneous ptychographic and XRF imaging should find its major application at diffraction-limited synchrotron light sources. An ultimately coherent flux will favour hybrid measurement schemes facilitating high-throughput characterization of a statistically-relevant population of samples. Supported by advances in instrumentation permitting artefact-free scanning of large areas and a minimal time overhead, the multimodal X-ray microscopy will enter a new era of becoming a routine method of choice in biomedical research.

## Methods

### Macrophages sample preparation

The studies were performed using primary macrophages generated from bone marrow myeloid progenitors isolated from C57Bl6 mice. Mice were bred and housed under specific pathogen free (SPF) conditions at the animal facility of the Research Centre Borstel-Leibniz Lung Centre. Usage of animals as bone marrow donors was approved by the Ministry of Energy, Agriculture, Environment, Nature and Digitization of the state Schleswig-Holstein, Germany, and by the ethical committee of University of Lübeck under the licence number V242-7224.123-3. All methods and experimental protocols were carried out according to relevant guidelines and regulations. We used murine-bone-marrow-derived macrophages as host cells. Firstly, the macrophages were deposited on a Si_3_N_4_ membrane coated with poly-L-lysine to facilitate cell adhesion. Subsequently, the monolayers of macrophages were treated with Fe_2_O_3_ nanocontainers in DMEM containing 10% FCS for 2 h at 37 °C/7% CO_2_ and fixed with 4% paraformaldehyde. Cells were visualized using a visible-light bright field imaging to identify the cells of interest for subsequent X-ray measurements. Afterwards, the cells were further fixed with 2.5% solution of glutaraldehyde in phosphate-buffered saline buffer. The cells were next postfixed with OsO_4_ and air-dried according to the protocol listed in Supplementary Method 4.

### Bone sample preparation

Bone samples were obtained from the tibia plateau of a 19-year-old male individual (surgical waste material), based on written consent of the patient. The study was approved by the local ethics committee (Hamburg Chamber of Physicians, WF-020/17). All experiments were performed in accordance with local guidelines and regulations. The bone material was fixed with neutrally buffered formalin within one hour from material collection to permit best results for studying bone matrix quality and cellular biology in combination. Once fixed, specimens were cut to a size of 1 cm^3^ using a diamond-coated band saw (Exakt, Norderstedt, DE) to allow for embedding in polymethylmetacrylate (PMMA) after dehydration in ascending grades of alcohol. The PMMA blocks including the specimen were cut using a microtome (Leica, Wetzlar, DE) to produce 4-μm-thin consecutive sections. The thin bone sections were deposited on Si_3_N_4_ membranes and air-dried.

### Experimental setup

The experiments were carried out at beamline P11 at the PETRA III synchrotron light source, DESY, Hamburg. P11 features an environment for high-resolution imaging and diffraction experiments^56^. We used a self-developed scanning transmission X-ray microscope at photon energies slightly above Fe K absorption edge (7.112 keV), which is explained in detail in Supplementary Method 5. Horizontal spatial coherence was enhanced by defocussing the X-ray beam with convexly bent horizontally deflecting X-ray mirrors that are components of P11 optics^57^.

**Figure 4:**
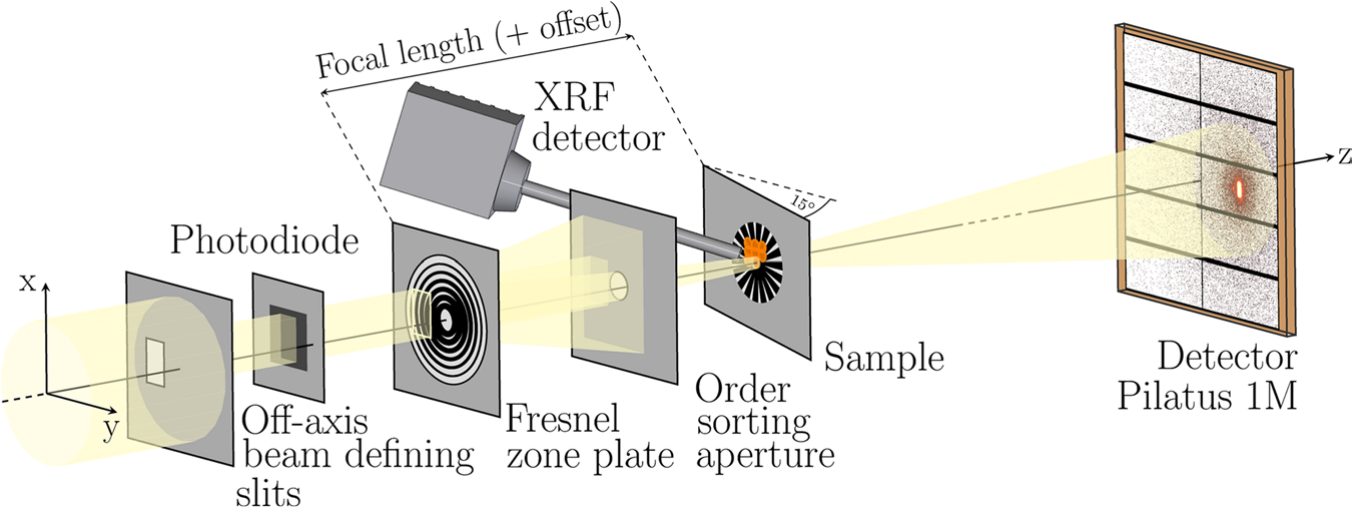
Scheme of the experimental setup for simultaneous ptychographic and X-ray fluorescence imaging at beamline P11 using a silicon photodiode for incoming flux measurement, an off-axis illuminated Fresnel zone plate, a silicon drift detector for X-ray fluorescence acquisition, and a single photon counting Pilatus pixel detector for recording coherent diffraction patterns. The sample was tilted by 15° with respect to the incoming X-ray beam for more efficient detection of XRF signal.

The coherent portion of X-ray beam was selected with 40 × 60-μm^2^ (h × v) off-axis beam defining slits (BDS) that partially illuminated a Fresnel zone plate (FZP) of a diameter of 200 μm and an outermost zone width of 30 nm, as shown in Fig. 4. The FZP holder was unmotorized and fixed at a constant position with respect to the setup base plate. A thin silicon photodiode was positioned between the BDS and the FZP to measure the incoming photon intensity. The first order focus was selected by an order sorting aperture of a diameter of 10 μm. The sample was attached to a kinematic base plate (Thor Labs, KBT1X1T) to enable fast and reproducible mounting. The specimen was placed in the vicinity of the back-focal plane and tilted by 15° to normal incidence to facilitate detection of XRF signal. Using an inhouse-developed flexure-based piezomotorized x/y stage, the sample was scanned across the beam. Its positions were controlled in both directions with laser interferometers (Attocube FPS3010). The scanning stage featured ±2-mm scanning ranges in both directions permitting batch-scanning over an entire Si_3_N_4_ membrane area without any secondary coarse positioning. Motion control was realized with GALIL DMC4080 Controllers. The continuous-motion scanning (fly-scan) was performed using carriage-return grid scan trajectory to significantly reduce the overhead time to less than 15% of the total scanning time. The far-field diffraction patterns were recorded with a single photon counting Pilatus pixel detector positioned downstream of the sample at a distance of 4.2 m. In the space between, a flight tube filled with helium was installed to reduce scattering and absorption of X-rays in air. Simultaneously, at each point of a scan, an XRF spectrum was acquired using an SDD detector Vortex-EM with a total active area of 50 μm^2^. Both detectors were externally triggered by a TTL signal generated by the Raspberry Pi Logic Controller (PiLC), multifunctional and customizable FPGA-based module for fast signal processing (Supplementary Method 5). Alongside trigger generation, the PiLC read out two encoder and two interferometer signals for both scan axes and corresponding values of the silicon diode at the same frame rate. At the end of each scan line, the data were retrieved from the PiLC buffer and flushed into an HDF5 metadata file together with XRF spectra and relevant beamline status parameters. Analysis tools used for spectra fitting and ptychographic reconstructions are described in Supplementary Method 6.

## Supplementary information

Supplementary Information.
