# Supplementary material for: Multimodal X-ray imaging of nanocontainer-treated macrophages and calcium distribution in the perilacunar bone matrix
Source: Sci Rep. 2020 Feb 4;10:1784. doi: 10.1038/s41598-020-58318-7 (PMC7000813; doi:10.1038/s41598-020-58318-7)
Supplement: Supplementary file 1 — Supplementary Information. [file 41598_2020_58318_MOESM1_ESM.pdf]

Supplementary material - Multimodal X-ray imaging of nanocontainer-treated macrophages and calcium distribution in the perilacunar bone matrix

Karolina Stachnik[1,2,*], Martin Warmer[1], Istvan Mohacsi[2], Vincent Hennicke[1,2], Pontus Fischer[1,2], Jan Meyer[1], Tobias Spitzbart[1], Miriam Barthelmess[1,2], Jacqueline Eich[3], Christian David[4], Claus Feldmann[5], Björn Busse[6], Katharina Jähn[6], Ulrich E. Schaible[3], and Alke Meents[1,2]

[1]DESY Photon Science, Deutsches Elektronen-Synchrotron DESY, 22607, Germany
[2]Center for Free-Electron Laser Science, Hamburg, 22607, Germany
[3]Research Center Borstel – Leibniz Lung Center, Borstel, 23845, Germany
[4]Paul Scherrer Institut, Villigen PSI, 5232, Switzerland
[5]Institute of Inorganic Chemistry, Karlsruhe Institute of Technology (KIT), Karlsruhe, 76131, Germany
[6]Department of Osteology and Biomechanics, University Medical Center Hamburg-Eppendorf, Hamburg, 22529, Germany
[*]karolina.stachnik@desy.de

Supplementary Method 1

Quantification of Fe signal (macrophages)

In this section we provide a detailed description on how we obtained iron areal mass maps from the raw iron distributions. For the quantification of an iron mass per unit area we used an iron film of a thickness of 470(2) nm deposited with electron-beam evaporation on a Si_3N_4 membrane. Repeated X-ray transmission measurements revealed the film density to be about 21% less than the tabulated bulk density. It may be a result of a different arrangement of Fe atoms in the deposited film than in a crystalline structure. An areal deposition mass of $2.827(17)\,\mu g\,mm^{-2}$ was then used for the areal mass calibration of the Fe maps. All Fe maps of the cells and the calibration standard were first normalized by the flux of the incoming X-ray beam (Si diode data) correcting for intensity fluctuations. Histograms of Fe counts from 5 independently measured calibration standard Fe maps were fitted with a Gaussian distribution and averaged yielding a mean of 3516(31) counts per resolution area. Combined with the areal mass of the calibration standard, a calibration coefficient of $0.8041(86)\,ng\,mm^{-2}\,count^{-1}$ was derived and applied to all Fe maps measured under the same experimental conditions. Ptychographic reconstructions overlaid with Fe distribution maps of all cells referenced in the main text are shown in Fig. S1.

The integrated Fe mass and the area of every nanocontainer agglomerate were calculated from all pixels within the agglomerate whose signal was greater than around $40\,ng\,mm^{-2}$ (50 counts). This threshold was chosen to exclude the background and the native cellular Fe levels.

The integrated Fe mass was calculated as a sum of the multiplications of Fe areal mass and the resolution area for every selected pixel, normalized by an overlap factor of 15.1. The resolution area ($525 \times 600\,nm^2$, h×v) was defined as the probe size ($400 \times 600\,nm^2$, h×v) augmented horizontally by the 125-nm step size due to continuous scanning. The overlap factor stemmed from the substantial oversampling of the Fe maps (necessary for ptychography), that would overestimate the integrated Fe mass. It was determined by simulating the intensity distribution within the entire scanned area, using the reconstructed probe profile and the relative sample-probe scan positions. 10 different areas of a size of the illuminating probe were isolated from such an intensity distribution. The total intensity accumulated within each of them was divided by the number of incident photons. Subsequently, 10 resulting values were averaged, yielding a mean overlap factor of 15.1.

The area of a nanocontainer agglomerate was calculated from the total number of pixels per agglomerate. Its uncertainty was estimated by approximating the agglomerate's shape to a circle.

The mass of a single nanocontainer of $1.13(36) \times 10^{-17}$ g was obtained assuming a hollow Fe_2O_3 sphere model with an inner diameter of 10(1) nm and an outer diameter of 18(1) nm[1]. Dividing the nanocontainer mass by the mass of Fe_2O_3 molecule, a number of Fe_2O_3 molecules per nanocontainer of 50000(11000) was estimated. The integrated Fe mass of each agglomerate was recalculated into the number of Fe_2O_3 molecules using tabulated atomic masses. By dividing the latter by the number of Fe_2O_3 per nanocontainer, we obtained the numbers of nanocontainers (NC) in each agglomerate. For example, the two nanocontainer agglomerates shown in Fig. 2a comprise 35300(7400) and 44400(9400) NC, for the left and the right agglomerate, respectively. The standard uncertainties of about 20% originate from uncertainties of the nanocontainer dimensions.

Supplementary Method 2

Spatial resolution analysis (bones)

While spatial resolutions of X-ray fluorescence mapping are inherently limited by the size of the X-ray beam footprint on the sample, the use of spatially coherent illumination and iterative phase retrieval instead of an objective lens makes ptychography surpass that limit. The ptychographic and X-ray fluorescence images of the human bone matrix shown in Fig. 3a and 3b are both rich in structural details and can be used to evaluate spatial resolution limits of both imaging techniques. For this purpose, we employed two distinct Fourier-transform-based methods suitable for each measurement.

Directional power spectral densities of X-ray fluorescence map

The resolution of the X-ray fluorescence calcium map (Fig. 3a) was derived from its power spectrum. We applied an edge-softening Tukey window function to the image and took the power of its discrete Fourier transform. The obtained two-dimensional power spectral density was then averaged vertically and horizontally to account, respectively, for different horizontal and vertical illumination sizes. Figure S2a shows two directionally averaged PSD curves whose intersections with the lines denoting twice the noise level[2] correspond to the half-period resolution limits of: 418 nm in the horizontal direction and 359 nm in the vertical direction. The obtained values remain in very good agreement with the probe size obtained by ptychographic reconstruction (200 nm$\times$400 nm, h$\times$v) broadened horizontally by the 200-nm step of the continuous scanning.

Fourier ring correlation of complementary ptychographic sub-datasets

We used the Fourier ring correlation (FRC) method[3] to estimate the spatial resolution of the bone matrix ptychographic reconstruction in Fig. 3b. FRC requires typically two independently acquired images. In the absence of a repeated measurement, phase reconstructions from two complementary sub-datasets can be correlated, providing a more conservative resolution estimation[4]. Here, two sub-datasets were created according to the following pattern: (1) for even scan lines: even-numbered diffraction patterns, for odd scan lines: odd-numbered diffraction patterns; (2) for even scan lines: odd-numbered diffraction patterns, for odd scan lines: even-numbered diffraction patterns. In reconstruction of both sub-datasets we used refinement of the scan positions to break the raster-scan periodicity, which alleviated grid-scan pathology artefacts. Solid blue line in Fig. S2b denotes the FRC between the two ptychographic sub-datasets. Its intersection with the 1/2-bit threshold line provides a spatial resolution limit of 65 nm.

Supplementary Method 3

Internalization of nanocontainer agglomerates in macrophages

In the absence of complete volumetric information, an additional verification of nanocontainers uptake is required. In a pilot study previous to the presented work, we used the scanning electron microscopy (SEM) to visualize the surface of the cell. We observed that the larger nanocontainer agglomerates were usually not internalized inside the cells and hence were visible in an SEM image. Figure S3a shows the ptychographic phase image of a representative macrophage treated with iron-oxide nanocontainers, while in Fig. S3b the ptychographic image was overlaid with the Fe distribution map. The Fe map allows to identify 5 nanocontainer agglomerates. A subsequently recorded SEM image of the same cell in Fig. S3c reveals that the largest agglomerate (dashed line) is located on the top of the cell. The remaining smaller agglomerates (solid line) are not visible and are hence supposed to be within the cytosol.

Supplementary Material 4

Sample post-preparation (macrophages)

In order to stabilize the sample and enhance the imaging contrast, the inactivated macrophages were stained with OsO_4 and, finally, dried in air according to the following protocol:

1) 10 min phosphate-buffered saline (PBS),
2) 10 min 1% OsO_4 in PBS,
3) dipping in water to remove bulk OsO_4,
4) 10 min washing in water,
5) ethanol (EtOH) gradient: 50/70/90/100/100% EtOH in water, 10 min each,
6) freon 113 gradient: 50/80/100/100% freon 113 in EtOH, 10 min each,
7) fresh freon 113, oven 40.5 °C, over night.

Supplementary Method 5

Experimental setup

Mechanical design

All measurements reported in this work were obtained using a proprietary in-air 2D scanning X-ray microscope featuring a long-scan-range flexure-based scanning unit. The microscope was installed on a 8-m-long granite block at beamline P11[5]. Figure S4a shows a rendered view of the mechanical design. The setup base plate (1) was attached to XYZ stepper motor translational Kohzu stages (not shown here) allowing to align the microscope with respect to the X-ray beam (denoted in red).

First element of the setup in direction of the beam path was a slit system (2) consisting of 4 independent tantalum blades controlled by piezo motors to select a coherent portion of the incident X-ray beam. The corresponding control software allowed for adjusting the slits center position and horizontal and vertical slit width. Second element in the beam was a 25-µm-thick silicon diode attached to a piezo motor stage (3), which was used to constantly monitor and record the flux of the incident X-ray beam.

Third element in the beam path were the Fresnel Zone Plates (FZPs) used for nano-focusing of the X-rays. The FZPs were deposited on a Si_3N_4 membrane which was glued to the downstream end of an aluminum tube (4). The tube was inserted into a solid support (5) and then fixed with two screws from the top (6). The position of the FZPs along the beam could be manually adjusted to a given FZP focal length (here approx. 35 mm). Figure S4b provides a magnified view of the setup in the sample area.

The FZPs were followed by a 10-µm-diameter order sorting aperture (OSA) manufactured from platinum, which was placed approximately 2 mm upstream of the sample. The OSA was glued to a laser-cut support manufactured from silicon (7, denoted in green). Using silicon as material for the OSA-support ensured no parasitic X-ray fluorescence background in the energy range of first-row transition metals, that would otherwise be caused by impurities in aluminum-alloy holders in close proximity of the sample (alloy EN AW-7075: Mn 0.3 Mg 2.1-2.9). The silicon support was directly attached to the aluminum arm (8) of an x,y piezomotor stage (9), without any magnetic coupling to avoid any long-term drifts. This x,y piezomotor stage was further mounted onto a stepper-motor Z translation stage for positioning of the OSA along the beam.

The specimens were prepared on Si_3N_4 membranes. For the measurements these membranes (denoted in blue) were glued to a sample kinematic base plate (10), which was magnetically mounted on the sample scanner (11). Main element of the sample scanning unit (12) is a 2D piezomotor-driven flexure stage. The flexure was manufactured from titanium alloy Ti-6Al-4V with electrical discharge machining (EDM). It is capable of scanning a $4{\times}4\,\mathrm{mm}^2$ field of view. The two scanning axes are each equipped with linear encoders and operated in closed loop.

For an independent positioning reference the scanner is further equipped with two interferometric sensors. For this, the sample scanner is equipped with two perpendicular mirrors which reflect signals of the interferometer lasers whose heads are denoted in yellow in Fig. S4. During the measurements we observed that the positions obtained from the incremental encoders were affected by intrinsic distortions on a micrometer level compared to the interferometer signals.

Both the OSA piezo motor stage (9) and the sample scanning unit (12) were fixed at a 15° angle with respect to the normal incidence to optimize the signal acquired with an XRF silicon drift detector, Vortex-EM (13). A tilt of the entire scanner unit ensured a constant position of the sample-beam interaction point along the beam direction and hence the same beam size over the entire scan range. The silicon OSA-support (7) was in turn positioned perpendicularly to the incident beam to avoid any beam clipping.

Alignment of the microscope required a CCD camera positioned within a short distance behind the sample scanner. It consisted of the following steps: (i) alignment of the FZP with the XYZ stepper-motor tower, (ii) alignment of the slits gap opening and position, the diode, and the OSA with respect to the FZP, (iii) inserting and aligning a test sample, (iv) optimizing the distance between the sample and the XRF detector.

Continuous-motion scanning with PiLC Trigger Generator

The Raspberry Pi Logic Control (PiLC) is a versatile solution to basic control and data acquisition problems occurring at synchrotron radiation experiments. It combines the data processing power of a FPGA chip and the high-level, user-friendly interface of an embedded Raspberry Pi. The FPGA guarantees the speed and the synchronicity of logical operations and of other data processing tasks. The scope of possible applications is limited only by the FPGA functionality.

The FPGA is connected to the experiment electronics by 16 lines that are configurable in terms of input/output and NIM/TTL. In addition, analog I/Os are also supported. The embedded PC runs under a standard operating system, Debian, and has the Tango control system installed.

The Tango servers have been developed for downloading the firmware and for giving access to FPGA registers, thereby allowing remote applications to read/write data from/to the FPGA and to control its operation. Figure S5 shows a PiLC unit with its front socket panel and interior electronic components.

In our experiment, the PiLC was utilized to implement the continuous-motion scanning. The PiLC TTL output was used to

send a 5 V standard trigger signal to synchronously acquire data with two detectors and to collect motor positions and incident flux data as described in the main text. The carriage-return continuous-motion scanning was implemented in the following manner: the sample was set at a position a few micrometers before the starting position. The offset allowed the horizontal motor to accelerate to the desired continuous-scanning speed. The starting horizontal position was fed into the PiLC, which would start sending the triggers only once the horizontal motor had reached that position. From that point on, the device would emit a given number of triggers at a specified frequency. The trigger signal length and period were decoupled and could be set up according to the specifications of the detectors. The motor would continue driving a few micrometers beyond the ending position so that its deceleration would not affect the data recorded at the end of the line. Afterwards, the motor would advance to the next line at a maximum design speed to reduce the time overhead.

Supplementary Method 6

Spectra fitting and ptychographic reconstructions

XRF spectra were batch-fitted using PyMca X-ray Fluorescence Toolkit[6]. The elemental maps were then corrected for scanning unit distortions with interferometer positions. Ptychographic datasets were reconstructed with the difference map algorithm[7] and three orthogonal probe modes[8], whose intensity fractions are listed in Table S1. Further increase of the probe modes number would not yield any qualitative improvement of the reconstructed images. Long-term source instability compromised the quality of some ptychographic reconstructions, which resulted in pronounced non-linear background phase offsets. In the case of single cells, empty image areas were used to obtain phase profiles for a global background correction. Alternatively, a representative affected scan (Fig. 1b) was split into several overlapping sub-scans, each updating the common object, but having independent probes. This approach diminished substantially the non-linear phase background.

Table S1. The intensity fractions of the orthogonalized probe modes obtained from 3-mode ptychographic reconstructions of the fly-scan datasets.

**Parameter**	**Macrophages**	**Bones**
Fly-scan speed [$\mu m\, s^{-1}$]	0.8	2.0
First mode fraction [%]	63.8	72.9
Second mode fraction [%]	22.0	16.7
Third mode fraction [%]	14.2	10.4

References

1. Leidinger, P. *et al.* Isoniazid@Fe_2O_3Nanocontainers and Their Antibacterial Effect on Tuberculosis Mycobacteria. *Angewandte Chemie Int. Ed.* **54**, 12597–12601 (2015).

2. Modregger, P., Lübbert, D., Schäfer, P. & Köhler, R. Spatial resolution in Bragg-magnified X-ray images as determined by Fourier analysis. *Phys. Status Solidi A* **204**, 2746–2752 (2007).

3. van Heel, M. & Schatz, M. Fourier shell correlation threshold criteria. *J. Struct. Biol.* **151**, 250–262 (2005).

4. Deng, J. *et al.* Simultaneous cryo X-ray ptychographic and fluorescence microscopy of green algae. *Proc. Natl. Acad. Sci.* **112**, 2314–2319 (2015).

5. Burkhardt, A. *et al.* Status of the crystallography beamlines at PETRA III. *The Eur. Phys. J. Plus* **131**, 56–9 (2016).

6. Solé, V. A., Papillon, E., Cotte, M., Walter, P. & Susini, J. A multiplatform code for the analysis of energy-dispersive X-ray fluorescence spectra. *Spectrochimica Acta Part B: At. Spectrosc.* **62**, 63–68 (2007).

7. Thibault, P., Dierolf, M., Bunk, O., Menzel, A. & Pfeiffer, F. Probe retrieval in ptychographic coherent diffractive imaging. *Ultramicroscopy* **109**, 338–343 (2009).

8. Thibault, P. & Menzel, A. Reconstructing state mixtures from diffraction measurements. *Nature* **494**, 68–71 (2013).

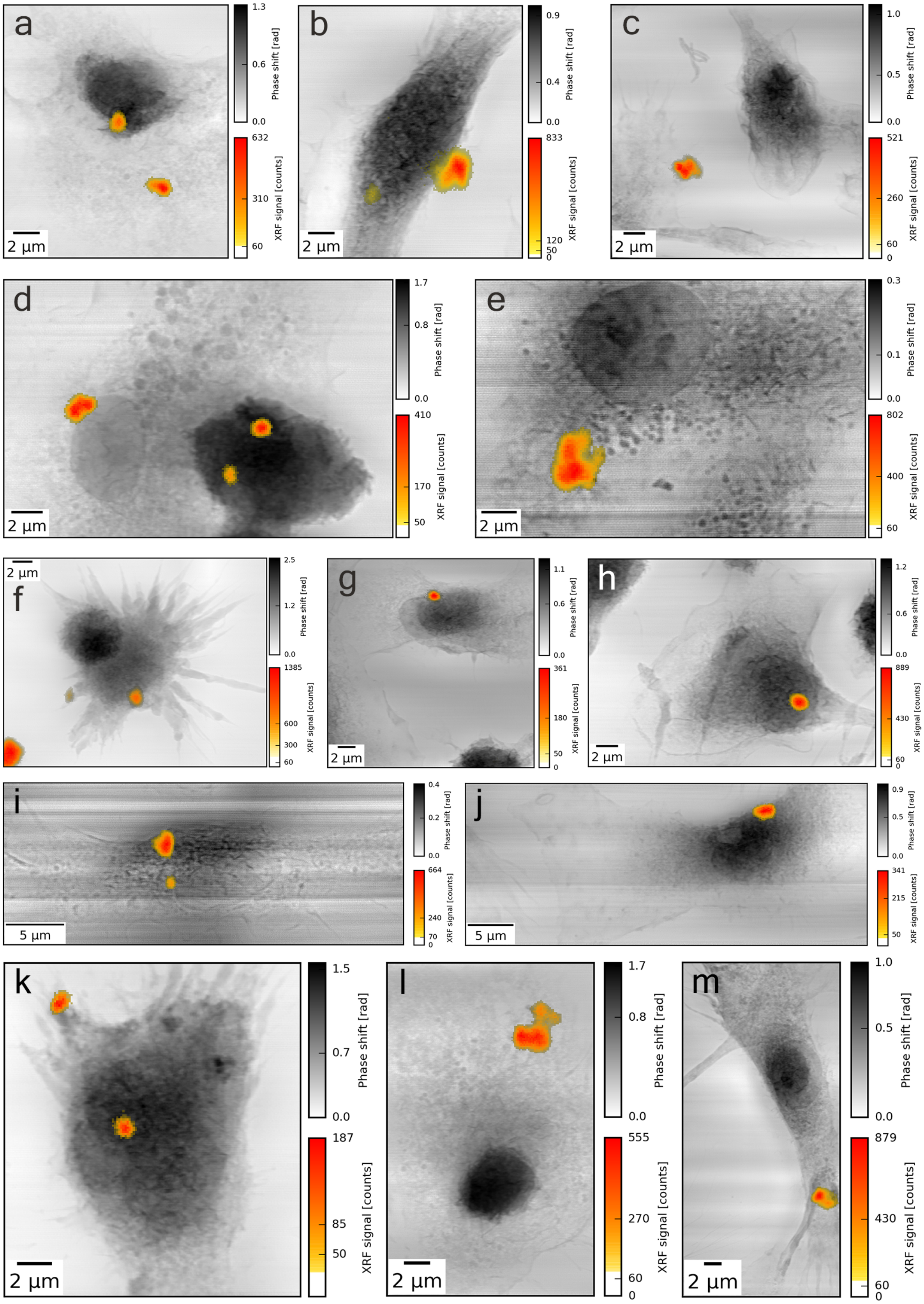

Figure S1. Ptychographic phase reconstructions overlaid with iron distribution maps of 13 macrophages treated with Fe_2O_3 nanocontainers measured under the same conditions. XRF signal scale denotes Fe K-line XRF yield.

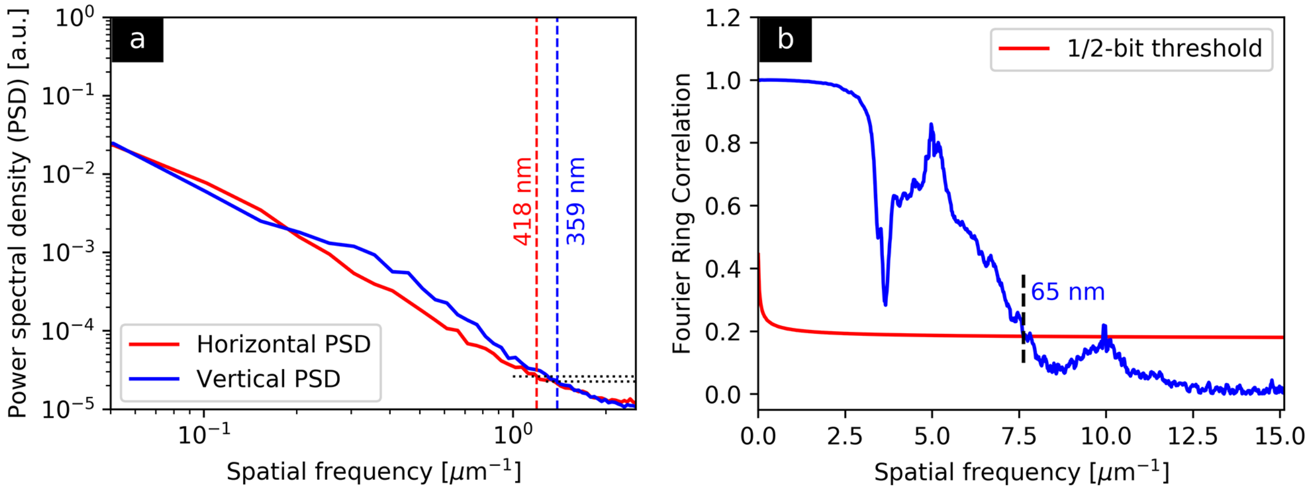

Figure S2. Spatial resolutions of the human bone section measurements. (**a**) presents horizontally- and vertically-averaged power spectral densities (PSD) of the raw Ca K-line map. The cross-sections of the PSD curves and twice the noise floor denotes half-period spatial resolutions of 418 nm×359 nm (h × v), respectively. Spatial resolution of the ptychographic phase image was evaluated by Fourier Ring Correlation (FRC), as shown in (**b**), between reconstructions of two sub-datasets being complementary halves of the whole set of diffraction patterns. The reconstructions involved position refinement to break the raster-scan periodicity. The cross-section of the FRC curve and 1/2-bit threshold criterion provides a conservative spatial resolution estimation of 65 nm.

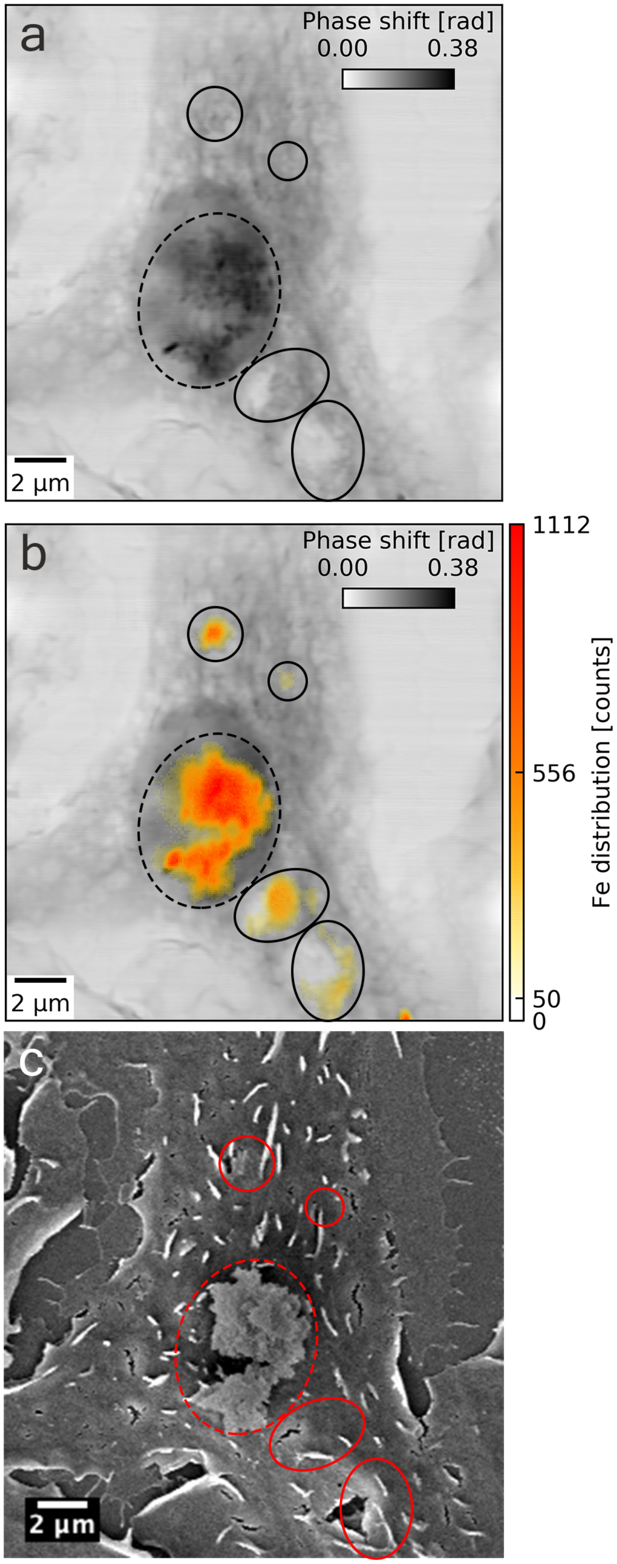

Figure S3. Verification of nanocontainer agglomerates internalization by scanning electron microscopy (SEM) based on a previous work. (**a**) shows the ptychographic phase image of an exemplary macrophage treated with iron-oxide nanocontainers. (**b**) shows the iron distribution map superimposed on the ptychographic image. 5 nanocontainer agglomerates can be identified. (**c**) shows an SEM image of the surface of the same cell. The largest agglomerate can be observed on the top of the cell (dashed line) whereas the remaining smaller agglomerates were internalized inside the cell.

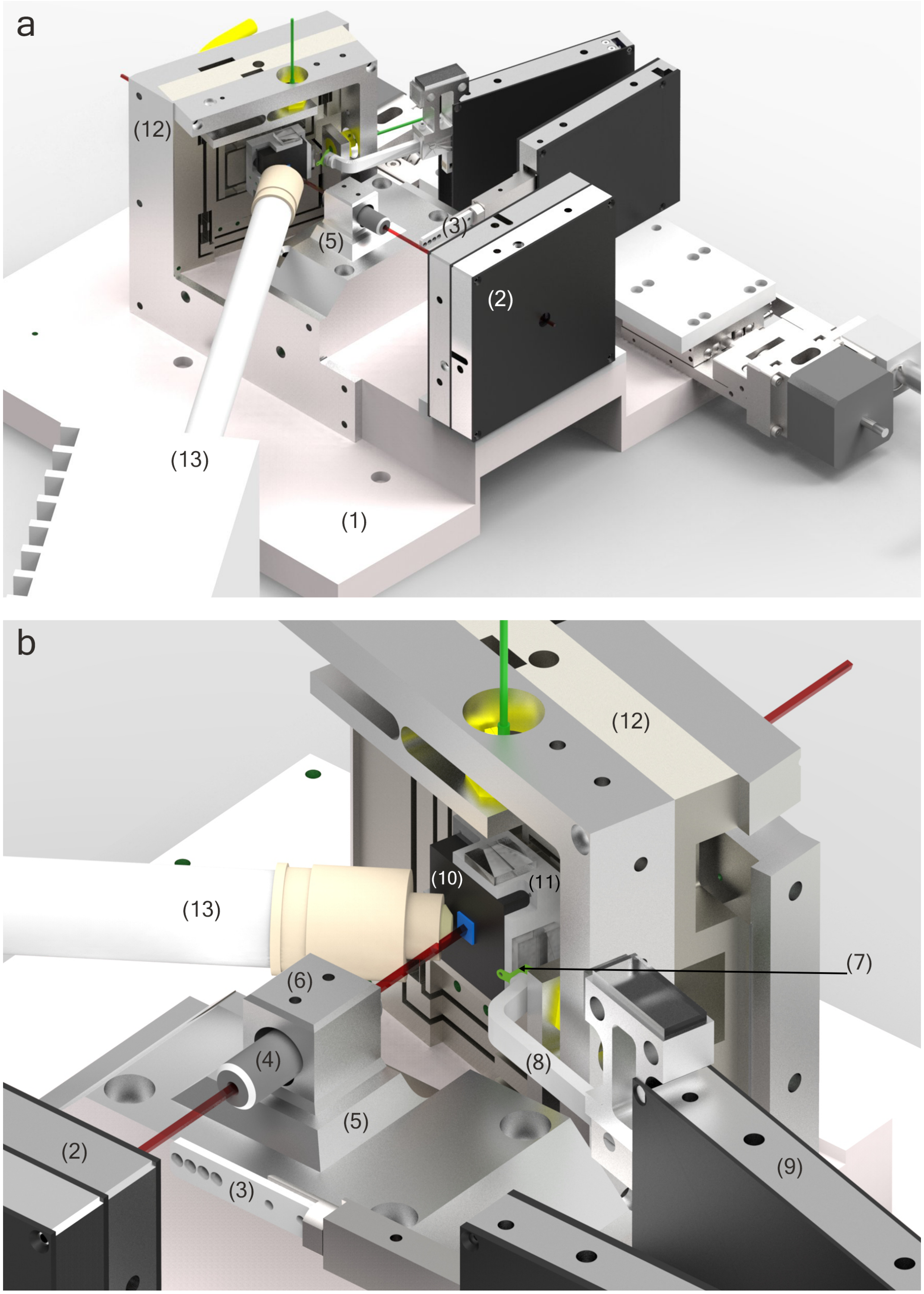

Figure S4. High-throughput scanning X-ray microscope (**a**) using a flexure-based scanner with a total travelling range of $4\times4\,mm^2$ (h×v) and an interferometric position control. (**b**) provides a closer view of the sample area. The X-ray beam was denoted in red. The setup components are: (1) aluminum-alloy base plate, (2) 4-blade slit system, (3) piezomotorized holder for the silicon diode, (4) Fresnel zone plate (FZP) tube, (5) FZP support, (6) two screws fixing the position of the FZP tube, (7) silicon support for an order sorting aperture (OSA), (8) piezomotorized arm, (9) OSA piezo motor, (10) sample kinematic base plate with a Si_3N_4 membrane carrying the specimen denoted in blue, (11) sample scanner, (12) flexure-based scanning unit, (13) silicon drift detector Vortex-EM. Two interferometer heads were denoted in yellow, whose signal was reflected by two perpendicular mirrors attached to the sample scanner.

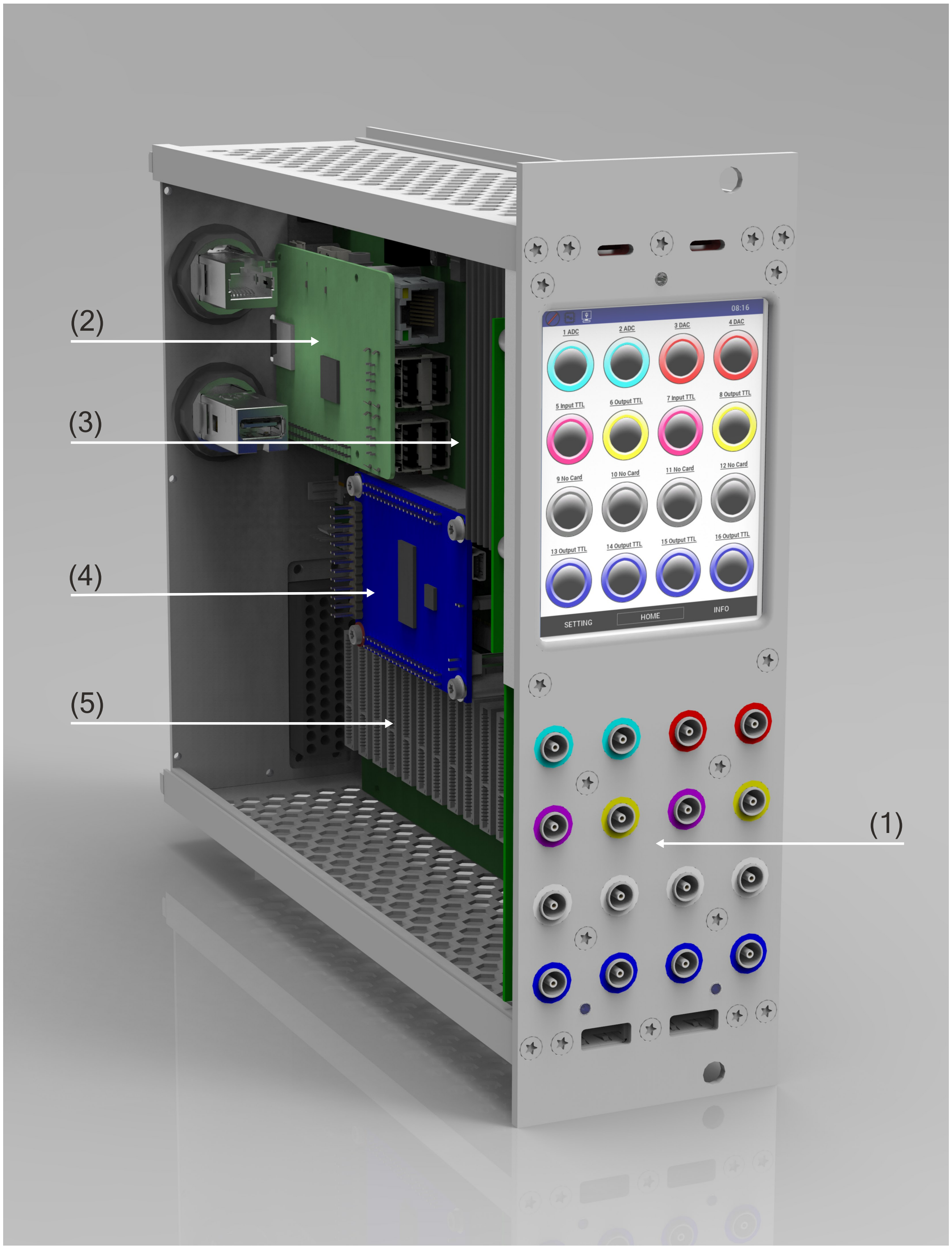

Figure S5. Raspberry Pi Logic Controller (PiLC) as a versatile device for basic control and data acquisition at synchrotron experiments. It is a multifunctional and customizable FPGA-based module capable of fast signal processing. The device consists of: (1) configurable digital and analog I/O ports, (2) Raspberry Pi with Ethernet interface, (3) mainboard, (4) Altera Cyclone IV FPGA, (5) 16 slots for I/O cards.
